# A Review of Adult Obesity Prevalence, Trends, Risk Factors, and Epidemiologic Methods in Kuwait

**DOI:** 10.1155/2013/378650

**Published:** 2013-12-17

**Authors:** Stalo Karageorgi, Osama Alsmadi, Kazem Behbehani

**Affiliations:** ^1^Genome Centre, Dasman Diabetes Institute, P.O. Box 1180, Dasman, 15462 Kuwait City, Kuwait; ^2^Dasman Diabetes Institute, P.O. Box 1180, Dasman 15462, Kuwait

## Abstract

*Objective*. Kuwait is among the countries with the highest obesity rates worldwide; however, little is known about the state of obesity epidemiology research in Kuwait. In this paper, we therefore review the findings and methodology of studies on the prevalence, trends and risk factors of obesity in Kuwait. *Methods*. The PubMed database was searched using the keyword combination: obesity and adults and Kuwait. Out of 111 articles, 39 remained after abstract review, and 18 were selected after full-text review. *Results*. The studies were all cross-sectional and published in the last fifteen years (1997–2012). The sample size ranged from 177 to 38,611 individuals. Only 30% of studies used random sampling. The prevalence (BMI ≥ 30) in studies with a nationally representative sample ranged from 24% to 48% overall and in adults >50 years was greater than 52%. Rates were significantly higher in women than those in men. Studies that examined trends showed an increase in obesity prevalence between 1980 and 2009. Multiple risk factors including sociocultural factors were investigated in the studies; however, factors were only crudely assessed. *Conclusion*. There is a need for future studies, particularly surveillance surveys and prospective cohort studies utilizing advanced methods, to monitor trends and to comprehensively assess the factors contributing to the obesity epidemic in Kuwait.

## 1. Introduction

Obesity prevalence rates have increased worldwide in the last three decades from 1980 to 2008, reaching a prevalence of 10–14% among the world's adult population in 2008 [1]. Even though obesity rates are higher in upper-middle income and high income countries [2], they are projected to increase rapidly in developing nations [3]. Body mass index (BMI) is the most common way of assessing obesity and is a measure of weight that adjusts for height [4] and correlates highly with body fatness [5]. Waist circumference (WC) on the other hand is a surrogate measure for abdominal fat and is suggested to be a better predictor for certain chronic diseases [4]. Obesity has been linked to a multitude of health conditions including diabetes, hypertension, ischaemic stroke and heart disease, different types of cancers, osteoarthritis, and reproductive conditions [4]. As a result, obesity is now among the leading factors for global morbidity and mortality and causes more global deaths than underweight [6].

**Figure 1:**
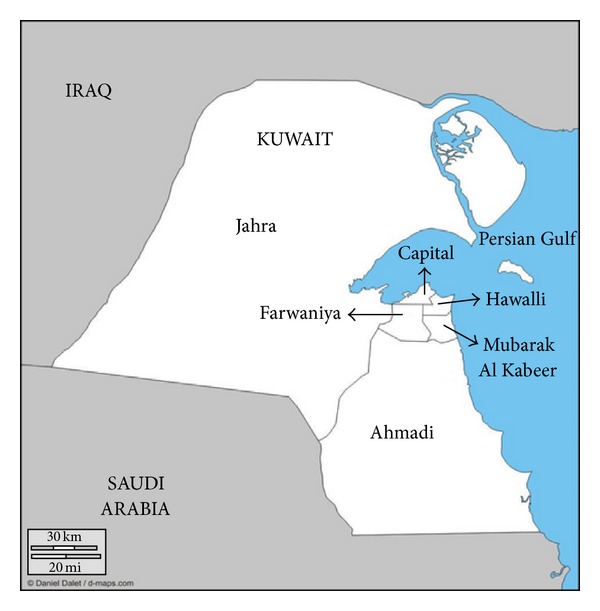
Map of the six governorates in Kuwait. Population by governorate in year 2012: Farwaniya (999,858), Hawalli (822,678), Ahmadi (738,023), Capital (514,198), Jahra (474,751), and Mubarak Al Kabeer (229,210). Population size represents both Kuwaiti and non-Kuwaiti nationals [11].

Economic growth, urbanization, and subsequent changes in lifestyle are among the factors driving the global obesity epidemic [7]. The rapid speed that the above factors advanced in countries of the Gulf region since the discovery of oil in the late 1930s may have exacerbated the obesity epidemic in these countries including Kuwait [8]. The state of Kuwait ranks in the top 7% of countries worldwide with the highest adult obesity prevalence rates according to the International Comparisons data from the WHO Global Infobase [9] and is in the top 3% of countries worldwide with the highest diabetes prevalence rates according to recent data from the International Diabetes Federation [10]. Kuwait, a high income economy, borders Iraq and Saudi Arabia, has a total population of 3,784,263, and is divided in six governorates (Figure 1). Only 32% of the total population are Kuwaitis with the remaining being Arabian (28%), Asian (38%), and other ethnicities (3%) [11].

The alarming levels of obesity and deleterious health consequences on the population of Kuwait led us to conduct a scientific review to evaluate the current state of obesity epidemiology research in Kuwait. Previous studies have reviewed obesity prevalence rates and causes in Arabic speaking countries [12], the Middle East [13], the Eastern Mediterranean [14, 15], and the Gulf region [16, 17]; however, none have focused exclusively on Kuwait. In this paper, we therefore summarize, classify, and synthesize available information on this subject. The purpose of this review is to conduct an exhaustive search and inclusion of obesity epidemiology studies in Kuwait in order to assess both research methodology and research findings. Studies are drawn from the literature reporting on the prevalence, trends, and risk factors associated with obesity in Kuwait.

## 2. Methods 

The PubMed database was searched for articles using the keyword combination: obesity AND adults AND Kuwait. Obesity was defined as BMI ≥ 30. Our search was not limited to year of publication and was limited to articles in English language. Publications were selected initially based on title and abstract review to include studies on the epidemiology of obesity and exclude studies not directly related to this subject. For the selected publications, we then conducted a full-text review and excluded studies with self-reported instead of measured weight and height, studies with redundant/overlapping data, and review studies that did not contribute new information through a meta-analysis. Among studies with data overlap, we used the study reporting the most detailed results, and for the review studies identified during the primary search, we screened their reference list for additional articles.

For each study, we retrieved information from the four study domains listed in Table 1. Finally, the reference list of retrieved articles and other science literature or public databases were searched for additional articles. Additional articles were also identified through personal communication. The last literature search was conducted on the 10th of February, 2013.

## 3. Results 

**Figure 2:**
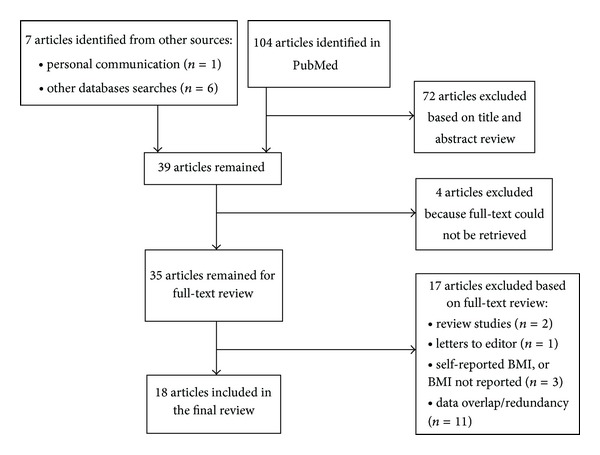
Flow chart for articles identification and selection.

The flow chart for article identification and selection is demonstrated in Figure 2. One hundred and four articles were identified in PubMed, and 32 articles [16, 17, 19–24, 26, 28, 30–51] remained after exclusion of nonepidemiologic studies based on title and abstract review. One additional article [52] was identified through personal communication and 5 articles [18, 25, 27, 29, 53] and one survey report [54] through other database searches leading to a total of 39 articles. Of the 39 epidemiology articles identified, 4 articles [40, 48–50] could not be retrieved and a total of 17 were excluded after full-text review for the following reasons. The excluded articles included two review studies [16, 17], one letter to editor [39], one study with self-reported BMI [36], one study that reported body weight but not height [51], one study that focused on anthropometric measures other than BMI [41], and eleven studies [37, 38, 42–47, 52–54] because of data overlap or redundancy. From among all studies with data overlap or redundancy, we selected the study reporting the most detailed results [19, 20, 22–24, 33, 35]. The selection process resulted in a final total number of 18 articles remaining for a thorough review [18–35] (Figure 2).

The data and information retrieved from the 18 reviewed studies are outlined in Table 2. These studies were published in the last fifteen years starting from 1997 through 2012. The data for the majority of the studies were collected in the 1993 through 2010 with the exception of one study [35] that additionally used data from the 1980s; however, there were four studies [21, 27, 28, 30] that did not report year of data collection. In studies reviewed, there was a lag of 2–5 years for publication since the last data collection. The time it took to recruit participants in most studies ranged from one day to three years and eight months although one survey study, called the Kuwait National Nutrition Surveillance System (KNNS) survey, ran for ten years and collected data biennially [22]. Five studies did not report the recruitment time period [20, 24, 27, 28, 30].

All eighteen studies reviewed used a cross-sectional study design. Five studies were national surveys (National Nutrition Survey (NNS) [19], Kuwait National Nutrition Surveillance System (KNNS) survey [22], WHO STEPS Survey [24], National Screening for Rheumatic Disorders Survey [32], and Nutrition Status Assessment of Adults Survey [35]), two studies were subnational based on selected areas of residence (governorates) [18, 34], six were hospital-based [21, 23, 26–29], and four were based at a college or university [20, 25, 33] or workplace [31]. A common eligibility criterion across the majority of the studies was Kuwaiti nationality; however, there were three studies which did not differentiate between Kuwaiti and non-Kuwaiti individuals during recruitment [25, 29, 31] and one study that purposely recruited both Arabs and South Asians [23]. With the exception of the KNNS survey that totaled 38,611 subjects over 10 years, the sample size of the remaining studies ranged from 177 to 7,609 subjects. The majority of studies covered a wide age range (on average from 19 through 70 years), but there were also three young adult studies (on average 17 through 25 years) [33], three studies with a shorter age span (19 through 49 years) [21, 27, 30] and one study on the elderly (≥50 years) [18]. Overall, the percentage of males in the studies ranged from 36% to 64%, despite two studies with higher percentage of 85% and 100% males [21, 31].

The sampling sources varied from colleges or universities (Public College of Basic Education, Kuwait College of Nursing, Kuwait University), to primary health care clinics, medical centers or private hospitals (Al Rashid Private General Hospital, Abdula Al Salem Health Center, Surra Family Practice Health Center, Qortuba Police Health Center). On the other hand, the KNNS study used mandatory health check-up points related to the Hajj pilgrimage, children's immunization, or government employment (Medical Council Center), and the social insurance registration center (Public Authority for Social Security Center) to recruit participants. Lastly, databases such as the civil identification database (Public Authority for Civil Information database), households, and selected areas of residence were used as sampling frames in some studies. Only five studies (30% of total) used random sampling methods (multistage cluster or stratified cluster sampling of households, and stratified random sampling of individuals or primary health care clinics) and all of these were national surveys except one study [18]. The remaining studies, including KNNS, used a nonrandom approach recruiting individuals on a volunteer basis at their chosen sampling source. Three [18, 19, 24] of the five random sampling studies reported a response rate ranging from 53% to 78% for households and 24% to 96% for individuals. Only three of the studies with nonrandom sampling reported a response rate that ranged from 85% to 95% among volunteer subjects. The remaining 11 out of the 18 studies did not report response rates.

The studies reviewed used face to face interview to collect data and only one study distributed a self-reported questionnaire to participants (Table 3). Out of the 17 studies that used face to face interview 11 studies explicitly stated the use of a questionnaire whereas the remaining 6 did not. The types of data collected in the reviewed studies are outlined for each study in Table 3 and summarized for all studies in Table 4. Some of the studies (*n* = 7/18) measured other anthropometric factors in addition to BMI including waist and hip circumference [19, 20, 23, 24, 26, 28, 30] while one of these studies additionally measured triceps and subscapular skinfold thickness [28] (Table S1 see supplementary material available online at http://dx.doi.org/10.1155/2013/378650). All studies used SPSS software to analyze their data. The statistical methods used in 10 out of the 18 studies included multivariate analysis statistical models (linear and/or logistic regression) while the remaining 8 studies used only bivariate analysis statistical tests (Student's *t*-test, chi-square test, *z*-test for proportions, ANOVA *F*-test, and ANCOVA test). The number of factors included in models to adjust for confounding ranged from zero to seventeen in the different studies. No justification was given in the studies to explain the choice of factors included in the model.

The overall prevalence of obesity (men and women combined) in the studies that reported overall prevalence rates ranged from 9% to 48%. When college-based studies are not taken into consideration, then the combined prevalence ranges from 20% to 48%. If only national studies are considered, the prevalence ranges from 24% to 48%. The majority of studies reported higher prevalence in women than men. Interestingly, in studies with young adult populations (17–25 years), the opposite was observed, higher prevalence in men than women. An increase with age was reported and among older age groups (>51 years), prevalence rates were greater than 52% for both sexes, greater than 39% for men, and greater than 67% for women in three national studies [19, 22, 24]. Finally, some studies additionally report a decrease in obesity rates in the very elderly (above 60 or 70 years) [18, 22].

Only two studies investigated temporal changes in obesity prevalence rates and reported a significant increase between 1980 and 1993 [35] and between 1998 and 2009 [22] (Table 3). About 70% of the studies examined obesity as the main outcome, whereas the remaining studies examined obesity as a risk factor for other health outcomes [20, 23, 26, 27, 30, 34] (Table S1). Among the risk factors reported to be associated with obesity in these studies were sociodemographic, socio-economic, sociocultural, lifestyle, dietary, and hereditary factors. The specific factors examined and the direction of the association reported are listed for each study in Table 1 and are categorized and summarized for all studies in Table 5. Among the studies that reported associations with health consequences, the health consequences examined and associated with obesity were health conditions such as diabetes, hypertension and osteoarthritis, and physiologic and biochemical outcomes (blood pressure, respiratory, blood lipids, and glucose measures).

Four studies had local and international author affiliations for first and last author [19, 21–23] while the remaining had local affiliations for both first and last author with the most common affiliation being Kuwait University. The journal impact factor for the studies reviewed ranged from 0.12 to 2.48 with the exception of one study with impact factor 7.82 [28] (Table S1).

## 4. Discussion 

This is the first review to evaluate obesity epidemiologic studies in Kuwait. In this review, we selected and extracted data from 18 studies according to specified criteria. This data was classified systematically in order to facilitate comparison of studies. Through this work, we were able to identify knowledge gaps and make recommendations for future research directions. This review was restricted to the adult population in Kuwait; however, there have been several informative studies on children. It was beyond the scope of this paper to review the findings on children in order to make possible a comprehensive review on existing adult studies.

The prevalence rates of obesity reported in national studies ranged from 24% to 48%. The prevalence increased in the last ten years and was significantly higher in women and older adults, indicating that these groups are particularly vulnerable. Not all results from the studies reviewed may be directly comparable because of differences in sampling procedures, age groups, and the year of data collection. Nevertheless, the careful extraction of vital information allows us to identify the factors that may have contributed to variation in results and compare studies which are more similar to each other. Furthermore, the exhaustive literature search and the wide inclusion criteria provide a clear understanding of the current state of obesity epidemiology research in Kuwait including methodology and findings.

The majority of epidemiologic studies reviewed used convenience sampling. Convenience sampling contrary to simple or other random sampling methods (stratified, systematic, cluster, or multistage sampling) may introduce bias because the sample may not be representative of the general population. However, there have been five national studies, four of which, used random sampling. The fifth study, KNNS, is the only one that provides detailed information on time trends; however, it did not use random sampling. Possible barriers to using random sampling by the majority of studies in Kuwait may have been requirement of formal access to lists of populations and low response rates among participants due to low levels of awareness, appreciation, and understanding of research. Furthermore, convenience sampling is easier, faster, and inexpensive and Kuwait's small population concentrated in few areas may have encouraged convenience sampling.

In relation to data collection strategies, the crude assessment of factors instead of a thorough and detailed assessment was common in current studies. In future studies, the assessment of different factors in questionnaires can be expanded to include multiple and detailed categories instead of binary categories. For example, information on the duration and intensity of several lifestyle factors including physical activity and smoking may be assessed in the future. The use of other data collection methods, besides face to face interview, such as computer based questionnaire, use of smartphone technologies, telephone interview, or other's can also be considered. In some studies statistical analyses, were limited to simple statistical tests such as chi-square tests and correlations rather than statistical modeling. The collection of detailed factors will also encourage the use of advanced statistical software and analysis that can provide robust findings adjusted for possible confounder's effect.

The results from existing studies on the correlates of obesity provide us with an overview of the risk factors that may be important contributors to obesity in Kuwait. For example, studies report on established factors such as age, gender, education, occupation, income status, physical activity, diet, and smoking but also bring to our attention the importance of sociocultural variables. The role of genetic susceptibility was recognized in existing studies but was only crudely assessed as family history of obesity. All existing studies used the cross-sectional study design and therefore are susceptible to reverse causation bias. Advanced studies, utilizing analytic epidemiologic designs such as prospective cohort, are needed to explore the etiology of “Kuwaiti” obesity in depth. In addition to the above factors, future studies may include investigation of newly emerging players in obesity such as gene-environment interactions, sleep deprivation, and developmental origins [5].

This review highlights the shortcomings of the methodologies used in obesity epidemiology research in Kuwait. Future studies should focus on overcoming these weaknesses by using state-of the-art methods. Priorities must include the design of systematic surveillance surveys to monitor trends and the design of prospective cohort studies with periodic data collections to examine obesity determinants and health consequences [5]. Surveillance monitoring in the United States is conducted by the National Health and Nutrition Examination Survey (NHANES). NHANES consists of cross-sectional surveys carried out annually since 1999 to monitor changes in obesity, physical activity, diet, and health outcomes. Around 7,000 individuals across different ages, ethnic backgrounds and income levels are randomly selected and interviewed every year, and collected data are made available to the research community for analysis. In Europe, the European Prospective Investigation into Cancer and Nutrition (EPIC) is an example of a prospective cohort study of half a million individuals in ten European countries followed every three to five years to update lifestyle and disease information. Similarly, in the Nurses' Health Study, 121,700 randomly selected nurses across the US have been followed every two years since 1976 with detailed questionnaires. Information collected from above studies has contributed to knowledge on determinants and health consequences of obesity. For the successful creation of similar studies in Kuwait, future design efforts should consider latest methods while also addressing possible challenges to quantitative research in the region related to systems support and sociocultural influences [55].

Anthropometric measures are the most common way of assessing adiposity. Future studies may incorporate use of new methods such as imaging techniques (dual-energy X-ray absorptiomentry, computed tomography, and magenetic resonance) that measure percentage of body fat and location of fat in tissues [5]. For example, body composition measurements have been extensively used in the Health, Aging and Body Composition (Health ABC) study, a prospective study of 3,000 individuals, to investigate the impact of changes in body composition on health of elderly. When focusing on metabolic consequences of obesity, which are highly prevalent in Kuwait, studies must include measures of WC, shown to better predict metabolic outcomes, or combine WC measurements with blood pressure, lipid, and glucose measurements, factors that describe the metabolic syndrome. In this review, less than half of the studies measured WC. Given the current controversy around the importance of fatness versus fitness, future studies must also include assessments of physical activity. Qualitative data from a study conducted in the UAE suggest that girls may be discouraged from exercising once they reach puberty [56]; this has important implications given that physically fit obese individuals may be at a lower risk than unfit obese for developing health outcomes. Finally, macrolevel factors including food subsidies policies in Kuwait and the role of food industry need to be further examined.

Improving the methodology of studies on trends, determinants and consequences of obesity is vital since results from these studies inform intervention and prevention strategies [5]. The true scale of the problem has not been thoroughly assessed by the existing studies. The primary factors responsible for placing Kuwait in the top 15 countries with the highest obesity prevalence out of 192 countries in the world [9], remain to be elucidated and addressed in prevention campaigns. The effective control and reduction of obesity in Kuwait will require a centralized campaign with policy strategies applied at multiple levels. Malik et al., in their recent review, thoroughly discuss examples of prevention programs implemented in other countries at the government, organization, community, and individual level [7].

In summary, we observed several studies published on the epidemiology of obesity in Kuwait; these were conducted in the last fifteen years and were all cross-sectional. Given the widespread and acknowledged problem of high obesity prevalence rates in Kuwait, we expect to see an increase in the number of studies in coming years. The recent establishment of Dasman Diabetes Institute by the state of Kuwait, a specialized research and treatment center on diabetes and related conditions, further highlights the urgent need to tackle these public health issues in Kuwait. Future research studies may focus on filling the gaps identified through this review and following a comprehensive approach to understanding and resolving the obesity epidemic in Kuwait.

## Supplementary Material

Table S1: Additional characteristics of adult obesity epidemiologic studies in Kuwait.

## Figures and Tables

**Table 1:** Information extracted from each reviewed study by study domain.

Study publication	Study design	Study analysis	Study results
First author	Year of survey	Statistical methods	Sample size (percent males)
Publication year	Recruitment period	Statistical model adjustment factors	Age range and/or mean age
Author affiliation*	Study design type	Statistical software	Ethnicity or nationality
Journal name*	Eligibility criteria		Obesity prevalence and/or mean BMI
Journal impact factor*	Sampling source/frame		Obesity correlates and/or trend
	Sampling method		
	Response rate		
	Data collection method		
	Type of collected data		
	Obesity category^∗#^		
	List of all collected variables reported*		
	Other anthropometric measures*		

*Data for these variables are listed in the supplementary table.

^#^Obesity category refers to whether obesity was examined as the main outcome or as a risk factor for other outcomes.

**Table 2:** Study design characteristics of adult obesity epidemiologic studies in Kuwait.

First author (publication year)	Year of survey (recruitment period)	Study design	Eligibility criteria	Sample size (% males) Age range (yrs) Ethnicity	Sampling source/frame	Sampling method (response rate)
Badr et al. (2012) [18]	2005-2006 (20 months)	Cross-sectional study (based on area of residence)	Kuwaiti, ≥50 yrs.	2,443 (39%) 50–70+ Kuwaiti	Kuwaiti households in Ahmadi and Capital governorates.	Multistage cluster sampling (78% of households). Households visited by interviewer (96% individuals).

Zaghloul et al. (2012) [19]	2008-2009 (16 months)	National cross-sectional survey (National Nutrition Survey (NNS))	Kuwaiti.	1,049 (45%) 19–86 yrs*Kuwaiti	Kuwaiti households from all six governorates.	Multistage cluster sampling (53% households) stratified by age and gender based on 2005 national census data. Subjects asked to attend interview at one of seven primary health clinics at various districts (24% of individuals).

Alattar et al. (2012) [20]	2009-2010 (not stated)	Cross-sectional study (college-based)	Kuwaiti, young adults, no current acute infection, not pregnant or diabetic, no diabetes inducing drugs.	484 (36%) 17–24 yrs Kuwaiti	Students attending the Public College for Basic Education between March 2009 and January 2010.	Nonrandom: volunteer students that learned about study through flyer advertisement and/or informational lectures about study (not stated).

Naser Al-Isa et al. (2011) [21]	Not stated (2 weeks)	Cross-sectional study (hospital-based)	Kuwaiti, males, ≥20 yrs.	464 (100%) 20–≥50 yrs Kuwaiti	One clinic in the capital.	Nonrandom: patient volunteers and those accompanying them (not stated).

Ahmed et al. (2011) [22]	1998–2009 (biennial measures)	National serial cross-sectional survey (Kuwait National Nutrition Surveillance System (KNNS))	Kuwaiti.	38,611 (45%) 20–69 yrs Kuwaiti	Medical Council Center mandatory health check-up point for government employment registration (80% of employed Kuwaitis work in public sector), Public Authority for Social Security Center for pension registration (all Kuwaitis receive retirement pension), mandatory health check-up point for Hajj Pilgrimage, parents accompanying children for mandatory immunizations at local health centers.	Nonrandom: volunteers among people attending mandatory health or social facilities (very low refusal rates).

Babusik and Duris (2010) [23]	2004–2007 (44 months)	Cross-sectional study (hospital-based)	Arab or South Asian ethnicity, absence of medical condition or drugs affecting outcome/exposures.	280 (64%) 18–69 yrs Arab: 51% South Asian: 49%	Al Rashid Private General Hospital.	Nonrandom: patient volunteers (not stated).

Al Rashdan and Al Nesef (2010) [24]	2006 (not stated)	National cross-sectional survey (WHO STEPS survey)	Kuwaiti, 20–65 yrs.	2,280 (40%) 20–65 yrs Kuwaiti	Public Authority of Civil Information (PACI) database used to randomly select sample of participants across 5 governorates in Kuwait.	Stratified random sampling: individuals asked to attend participating primary health care clinic for interview (78%).

Al-Kandari et al. (2008) [25]	2005 (one day)	Cross-sectional study (college-based)	Kuwait College of Nursing students.	202 (28%) 17–35 yrs Kuwaiti: 43% GCC: 29% Other Arab: 20% Non-Arab: 8.3%	All associate degree students enrolled in 2nd semester of 2004-5 (total of 350 students) at Kuwait College of Nursing.	Nonrandom: students in class on a specific day who agreed to participate (88%).

Al-Bader et al. (2008) [26]	2004–2006 (2 years)	Cross-sectional study (hospital-based)	Kuwaiti, adults, FEV1 > 80%, absence of smoking, pulmonary, cardiac, neurological, spine diseases.	380 (53%) 20–65 yrs Kuwaiti	Six medical centers covering all six governorates in Kuwait.	Nonrandom: patient volunteers (not stated).

Al Orifan et al. (2007) [27]	Not stated (not stated)	Cross-sectional study (hospital-based)	Kuwaiti, adults, absence of chronic disease, not pregnant.	296 (60%) 20–44 yrs Kuwaiti	Qortuba Police Health Center, Abdulla Al Salem Health Center.	Nonrandom convenience sample: volunteer subjects attending routine health check-up (not stated).

Al-Kandari (2006) [28]	Not stated (not stated)	Cross-sectional study (hospital-based)	Kuwaiti, adults.	424 (50%) 21–77 yrs Kuwaiti	Primary health care clinics or home interviews.	Nonrandom opportunistic sample: volunteers from six governorates of Kuwait (not stated).

Al-Assomi et al. (2005) [29]	2002-2003 (13 months)	Cross-sectional study (hospital-based)	>30 years.	597 (41%) 30–70+ yrs Kuwaiti: 77% Non-Kuwaiti: 23%	Surra Family Practice Health Center.	Nonrandom: volunteer Surra district residents that learned about study through posters, brochures, and two open days for raising awareness were given appointment for interview at the clinic (not stated).

Al-Shayji and Akanji (2004) [30]	Not stated (not stated)	Cross-sectional study	Kuwaiti, <50 yrs, no prior chronic illness, not pregnant.	177 (41%) 18–50 yrs Kuwaiti	A wide section of Kuwaiti population.	Nonrandom: volunteers who found out study through advertisement (not stated).

Al-Asi (2003) [31]	1999-2000 (18 months)	Cross-sectional study (company based)	Kuwait Oil Company employees.	3,282 (85%) Median age: 40 yrs Kuwaiti: 62% Non-Kuwaiti: 38%	All full-time employees due for their periodic medical examination between June 1999 and December 2000.	Nonrandom: full-time company employees due for their medical examination who agreed to participate (95%).

Olusi et al. (2003) [32]	2001 (9 months)	National cross-sectional survey (rheumatic disorders' prevalence survey)	Kuwaiti, adults > 15 yrs.	7,609 (52%) 15–84 yrs Kuwaiti	Kuwaiti households.	Randomly chosen households from all six governorates in Kuwait according to population size of each governorate. Households visited by interviewer (not stated).

Al-Isa (1999) [33]	1997 (5 days)	Cross-sectional study (university-based)	Kuwait University students.	842 (46%) <18–≥23 yrs Not stated	All male and female students coming in the first 5 days of registration for the 1997 fall semester at Kuwait University.	Nonrandom: students coming in to register who volunteered to participate in study (85%).

Abdella et al. (1998) [34]	1995-1996 (9 months)	Cross-sectional study (based on area of residence)	Kuwaiti, >20 yrs.	3,003 (37%) 20–≥60 yrs Kuwaiti	Hawalli and Capital governorate.	Nonrandom: volunteer subjects that learned about study through a publicity campaign (newspaper, radio, TV, brochures, posters at homes, supermarkets and post offices) were asked to attend the primary health care center in their area of residence (response lower in men).

Al-Isa (1997) [35]	1980-1981 (not stated) 1993-1994 (12 months)	1980: National cross-sectional survey (Nutrition Status Assessment of Adults Survey) 1993: Cross-sectional study (hospital-based)	Kuwaiti, adults.	1980: 2,067 (43%) 18–≥60 yrs Kuwaiti 1993: 3,435 (50%) 18–≥60 yrs Kuwaiti	Primary health care clinics.	1980 sample: stratified random sampling of 17 primary health care clinics in 5 governorates. Sample stratified by gender according to population gender ratio (not stated). 1993 sample: volunteer patients and those accompanying the patients attending 6 randomly selected primary health care clinics in 5 governorates of Kuwait (85%).

*Study also recruited individuals from 3–18 yrs of age; however, only data from adults from this study are presented in this review.

Studies are sorted by publication year.

**Table 3:** Data collection, data analysis, and results of adult obesity epidemiologic studies in Kuwait.

First author (publication year)	Data collection method (type of collected data reported)	Statistical methods	Statistical model adjustment factors	Obesity prevalence (%) or mean BMI	Obesity correlates and/or trend
Badr et al. (2012) [18]	Face to face interview with questionnaire (sociodemographic, socioeconomic, anthropometric, medical history, psychological).	Chi-square test, Student's *t*-test, multivariate logistic regression.	Age, sex, marital status, education, household income, cultural background (Bedouin, non-Bedouin self-identity).	All: 46%. Men: 30%. Women: 56%.	Female gender (+), being married (+), younger age among the ≥50 yrs population for example, 50–59 yrs versus 70+ (+), diabetes, hypertension and osteoarthritis (+), high depressive symptoms score in men (−)^$^.

Zaghloul et al. (2012) [19]	Face to face interview with questionnaire (sociodemographic, socioeconomic, anthropometric, dietary).	Student's *t*-test, ANOVA test.	No model used.	All: 46%*. Age group 19–50 yrs: all 41%*, men 29%, women 50%. Age group ≥ 51 yrs: all 57%*, men 42%, women 70%.	Not investigated.

Alattar et al. (2012) [20]	Face to face interview (sociodemographic, anthropometric, medical history, physiologic, biochemical, lifestyle).	Chi-square test.	No model used.	All: 20%. Men: 31%. Women: 14%. Mean BMI: all 26.	Not investigated.

Naser Al-Isa et al. (2011) [21]	Face to face interview (sociodemographic, socioeconomic, anthropometric, lifestyle, medical history).	Chi-square test, multivariate logistic regression.	Age, dental health status, chronic disease, number of obese brothers, number of obese relatives, parental obesity, wife's education, last GPA, high school GPA, monthly family income, physical activity, practice sport ( hours/week), practice sports (months/year), health status, dieting, feeling tired, need special nutrition program^#^.	Men: 20%. Men age group ≥ 50 yrs: 28%.	Age (+), treated dental status versus healthy (−), having chronic disease (+), number of obese brothers (+), number of obese relatives (+), parental obesity (+), educated wife (−), low high school GPA (+), high family income (+), physical activity (−), sports practice (−), poor health status (+), feeling tired (+), need for special nutrition program (+).

Ahmed et al. (2011) [22]	Face to face interview (sociodemographic, socioeconomic, anthropometric, lifestyle).	Mann-Whitney *U*-test, *Z*-test, multivariate linear regression, multivariate logistic regression.	Age and education when examining time trend. Age, education, exercise, smoking when examining risk factors.	All study years*: all 37%, men 32%, women 41%. Age group ≥ 50 yrs: all 52%, men 39%, women 67%. By study year: men: 1998—23%, 2000—31%, 2002—32%, 2004—39%, 2006—37%, 2008—34%. Women: 1998—28%, 2000—33%, 2002—49%, 2004—49%, 2006—49%, 2008—43%.	Trend: positive between 1998 and 2009 (peak in 2004). Risk factors: age (+), high education in women (−), high education in men (+), smoking and exercise in men (−).

Babusik and Duris (2010) [23]	Face to face interview (sociodemographic, anthropometric, biochemical).	*T*-test, Pearson correlation, multivariate linear regression.	Age, gender, nationality.	Arabs mean BMI: men 32, women 36. South Asians mean BMI: men 26, women 29.	Arab ethnicity versus south asian (+)^$^, age (+)^$^, HDL (−), TC/HDL ratio (+), triglycerides (+).

Al Rashdan and Al Nesef (2010) [24]	Face to face interview with questionnaire (sociodemographic, anthropometric, physiologic, biochemical).	Chi-square test, *Z*-test for proportion.	No model used.	All: 48%. Men: 39%. Women: 53%. Age group 20–24 yrs: men 23%, women 21%. Age group 55–65 yrs: men 43%, women 77%.	Age (+), female gender (+), mean SBP and DBP (+), total cholesterol (+), HDL (−), LDL (+), triglycerides (+), fasting glucose (+), HbA1c (+), waist circumference (+).

Al-Kandari et al. (2008) [25]	Self-reported questionnaire (sociodemographic, socioeconomic, anthropometric, health-promoting behavior).	ANOVA *F*-test.	No model used.	All: 12%. Men: 14%. Women: 11%. Kuwaiti: 8.3%. GCC: 1.4%. Other Arabs: 2%. Non-Arab: 0.5% Mean BMI: all 24, men 25, women 24.	Kuwaiti nationality (+), age (+), married (+), health promotion lifestyle score (−).

Al-Bader et al. (2008) [26]	Face to face interview (sociodemographic, anthropometric, spirometry).	ANOVA, *t*-test, linear regression.	No variables used to adjust for confounding.	Mean BMI: men 28, women 29.	Forced expiratory volume in 1 second (−), forced vital capacity (−).

Al Orifan et al. (2007) [27]	Face to face interview (sociodemographic, anthropometric, lifestyle, biochemical, physiologic).	Chi-square test, *t*-test, multivariate logistic regression.	Age, gender, systolic blood pressure or diastolic blood pressure, fasting blood sugar, triglycerides, total cholesterol or LDL and HDL cholesterol.	All: 42%	Female gender (+), impaired fasting blood sugar (+), prehypertension (+), high total cholesterol (+), high HDL (+), low LDL (+).

Al-Kandari (2006) [28]	Face to face interview with questionnaire (sociodemographic, Socioeconomic, sociocultural, anthropometric, lifestyle).	Correlation, multivariate linear regression.	Level of education, age, SES, number of families living in the same household, Number of times per week eating at restaurants, degree of preferring salt in food^#^.	All: 41%. Men: 39%. Women: 42%. Age group ≥ 50 yrs*: all 60%, men 43%, women 80%.	Level of education (−), age (+), SES (−), number of families living in the same household (+), Number of times per week eating at restaurants (+), degree of preferring salt in food (+), general physical activity (−)^$^, physical activity during work (−)^$^, number of relatives living in the same household (+)^$^, degree of religiosity (+)^$^, having a cook (+)^$^.

Al-Assomi et al. (2005) [29]	Face to face interview with questionnaire (sociodemographic, Socioeconomic, anthropometric, lifestyle, medical history, physiologic, biochemical).	Chi-square test.	No model used.	All: 44%. Men: 31%. Women: 53%. Kuwaiti: 50%. Non-Kuwaiti: 28%.	Hypertension (+), cholesterol (+), diabetes (+).

Al-Shayji and Akanji (2004) [30]	Face to face interview with questionnaire (sociodemographic, anthropometric, lifestyle, medical history, reproductive, physiologic, biochemical).	ANOVA, Student's *t*-test, ANCOVA, chi-square test.	No model used.	All: 20%. Men: 13%. Women: 25%. Mean BMI: all 26, men 25, women 26,	Glucose (+), LDL (+), apo B (+), urate (+), mean BP (+), triglycerides (+), insulin (+), insulin/glucose ratio (+).

Al-Asi (2003) [31]	Face to face interview with questionnaire (sociodemographic, anthropometric, lifestyle, medical history, physiologic).	Chi-square test.	No model used.	All: 27%. Kuwaiti: 32%. Non-Kuwaiti: 19%.	Kuwaiti nationality (+), field work versus office work (+), physical activity (−), diabetes (+), hypertension (+).

Olusi et al. (2003) [32]	Face to face interview with questionnaire (sociodemographic, Socioeconomic, anthropometric, medical history, lifestyle, and biochemical).	*T*-test, ANOVA.	No model used.	All: 24%. Men: 18%. Women: 30%.	Female gender (+).

Al-Isa A. (1999) [33]	Face to face interview with questionnaire (sociodemographic, Socioeconomic, anthropometric, medical history, lifestyle).	Chi-square test, multivariate logistic regression.	Gender, age, marital status, obesity among parents, dieting, last health check-up, year of study at college^#^.	All: 9%. Men: 11%. Women: 8%.	Male gender (+), maternal obesity (+), dieting (+), last health check-up a year ago versus do not recall last health check-up (−), second year of study versus first year of study (−), number of brothers/sisters (+)^$^, low high-school GPA (+)^$^, number of regular meals eaten (+)^$^.

Abdella et al. (1998) [34]	Face to face interview with questionnaire (sociodemographic, Socioeconomic, anthropometric, medical history, biochemical, physiologic, lifestyle).	ANOVA, multivariate linear regression, multivariate logistic regression.	Age, blood pressure, family history of type 2 diabetes, cholesterol, triglycerides, sex, exercise.	All: 40%	Type 2 diabetes (+), fasting plasma glucose (+).

Al-Isa. (1997) [35]	1980: not stated (sociodemographic, anthropometric). 1993: face to face interview (sociodemographic, Socioeconomic, anthropometric).	Chi-square test, *t*-test, multivariate linear regression, multivariate logistic regression.	Study period, age, region, education, marital status, occupation.	1980: men 59%, women 32%. 1990: men 73%, women 41%.	Study period 1993-94 (+), age (+), Ahmadi region (+), high education in men (+), married, widowed or divorced in women (+), working in women (+).

^$^Where multivariate results were not available for a specific variable the bivariate results are reported.

^#^Model adjustment factors not explicitly stated in paper text but above factors were included in the multivariate analysis table in paper.

*If parameters were not directly provided, these were estimated from numbers provided in study.

**Table 4:** Types of data collected in reviewed studies.

Sociodemographic	Dietary
Socioeconomic	Hereditary/family history
Sociocultural	Medical history
Behavioral	Anthropometric
Psychological	Physiologic
Lifestyle	Biochemical
Reproductive	

**Table 5:** Obesity risk factors* reported in reviewed studies.

Sociodemographic	Socioeconomic	Sociocultural	Lifestyle	Dietary	Hereditary
Female gender	Low high-school GPA	Number of families living in same household	Physical activity (−)	Number of regular meals eaten daily	Number of obese brothers
Male gender in college students	Education level (−)	Number of relatives living in same household	Practice sports (−)	Number of times per week eating at restaurants	Number of obese relatives
Age	High education in men	Number of siblings	Exercise in men (−)	Degree preferring salt in food	Paternal obesity
Being married	High education in women (−)	Degree of religiosity	Smoking in men (−)	Need for special nutrition program	Maternal obesity
Kuwaiti versus not	Educated wife (−)		Treated dental health status (−)	Dieting	Arab versus South Asian ethnicity
Ahmadi governorate versus capital	Working women		Recent health check-up (−)		
	Field versus office work		Health promoting lifestyle score (−)		
	High family income		High depression score in men (−)		
	Socioeconomic status (−)				
	Employing household cook				

*Factors are positively associated with obesity unless inverse association (−) is noted in the parenthesis next to risk factor.

## References

[1] Finucane MM, Stevens GA, Cowan MJ (2011). National, regional, and global trends in body-mass index since 1980: systematic analysis of health examination surveys and epidemiological studies with 960 country-years and 9*·*1 million participants. *The Lancet*.

[2] (2011). Global status report on noncommunicable diseases 2010.

[3] Kelly T, Yang W, Chen C-S, Reynolds K, He J (2008). Global burden of obesity in 2005 and projections to 2030. *International Journal of Obesity*.

[4] Remington PL, Brwonson RC, Wegner MV (2010). *Chronic Disease Epidemiology and Control*.

[5] Hu F (2008). *Obesity Epidemiology*.

[6] (2009). Global health risks: mortality and burden of disease attributable to selected major risks.

[7] Malik VS, Willett WC, Hu FB (2013). Global obesity: trends, risk factors and policy implications. *Nature Reviews Endocrinology*.

[8] Guy GW, Nunn AVW, Thomas LE, Bell JD (2009). Obesity, diabetes and longevity in the gulf: is there a gulf metabolic syndrome?. *International Journal of Diabetes Mellitus*.

[9] WHO Global info base international comparisons. https://apps.who.int/infobase/Comparisons.aspx.

[10] International Diabetes Federation *IDF Diabetes Atlas*.

[11] The public authority for civil information statistics http://stat.paci.gov.kw/englishreports/.

[12] Badran M, Laher I (2011). Obesity in arabic-speaking countries. *Journal of Obesity*.

[13] Motlagh B, O’Donnell M, Yusuf S (2009). Prevalence of cardiovascular risk factors in the middle east: a systematic review. *European Journal of Cardiovascular Prevention and Rehabilitation*.

[14] Musaiger AO (2011). Overweight and obesity in eastern mediterranean region: prevalence and possible causes. *Journal of Obesity*.

[15] Musaiger AO, Al-Hazzaa HM (2012). Prevalence and risk factors associated with nutrition-related noncommunicable diseases in the eastern mediterranean region. *International Journal of General Medicine*.

[16] Ng SW, Zaghloul S, Ali HI, Harrison G, Popkin BM (2011). The prevalence and trends of overweight, obesity and nutrition-related non-communicable diseases in the arabian gulf states. *Obesity Reviews*.

[17] Alhyas L, McKay A, Balasanthiran A, Majeed A (2011). Prevalences of overweight, obesity, hyperglycaemia, hypertension and dyslipidaemia in the gulf: systematic review. *JRSM Short Reports*.

[18] Badr HE, Shah NM, Shah MA (2013). Obesity among Kuwaitis aged 50 years or older: prevalence, correlates and comorbidities. *Gerontologist*.

[19] Zaghloul S, Al-Hooti SN, Al-Hamad N (2013). Evidence for nutrition transition in Kuwait: over-consumption of macronutrients and obesity. *Public Health Nutrition*.

[20] Alattar A, Al-Majed H, Almuaili T, Almutairi O, Shaghouli A, Altorah W (2011). Prevalence of impaired glucose regulation in asymptomatic Kuwaiti young adults. *Medical Principles and Practice*.

[21] Naser Al-Isa A, Campbell J, Desapriya E (2011). Factors associated with overweight and obesity among Kuwaiti men. *Asia-Pacific Journal of Public Health*.

[22] Ahmed F, Waslien C, Al-Sumaie MA, Prakash P (2012). Secular trends and risk factors of overweight and obesity among Kuwaiti adults: national nutrition surveillance system data from 1998 to 2009. *Public Health Nutrition*.

[23] Babusik P, Duris I (2010). Comparison of obesity and its relationship to some metabolic risk factors of atherosclerosis in arabs and south asians in Kuwait. *Medical Principles and Practice*.

[24] Al Rashdan I, Al Nesef Y (2010). Prevalence of overweight, obesity, and metabolic syndrome among adult Kuwaitis: results from community-based national survey. *Angiology*.

[25] Al-Kandari F, Vidal VL, Thomas D (2008). Health-promoting lifestyle and body mass index among college of nursing students in Kuwait: a correlational study. *Nursing and Health Sciences*.

[26] Al-Bader WR, Ramadan J, Nasr-Eldin A, Barac-Nieto M (2008). Pulmonary ventilatory functions and obesity in Kuwait. *Medical Principles and Practice*.

[27] Al Orifan FH, Badr HE, Abdul Sabour Se’adah M, Elias Khadadah K, Al Kordi B, Abass A (2007). Obesity and cardiovascular risk factor in Kuwaiti adults. *Kuwait Medical Journal*.

[28] Al-Kandari YY (2006). Prevalence of obesity in Kuwait and its relation to sociocultural variables. *Obesity Reviews*.

[29] Al-Assomi F, Al-Kandari S, Al-Wadaani D, Thalib L (2005). Prevalence of cardiovascular risk factors amongst the population of Surra, Kuwait. *Journal of the Bahrain Medical Society*.

[30] Al-Shayji IAR, Akanji AO (2004). Obesity indices and major components of metabolic syndrome in young adult arab subjects. *Annals of Nutrition and Metabolism*.

[31] Al-Asi T (2003). Overweight and obesity among Kuwait oil company employees: a cross-sectional study. *Occupational Medicine*.

[32] Olusi SO, Al-Awadi AM, Abraham M (2003). Baseline population survey data on the prevalence of risk factors for coronary artery disease among Kuwaitis aged 15 years and older. *Annals of Saudi Medicine*.

[33] Al-Isa AN (1999). Obesity among Kuwait university students: an explorative study. *Journal of The Royal Society for the Promotion of Health*.

[34] Abdella N, Al Arouj M, Al Nakhi A, Al Assoussi A, Moussa M (1998). Non-insulin-dependent diabetes in Kuwait: prevalence rates and associated risk factors. *Diabetes Research and Clinical Practice*.

[35] Al-Isa AN (1997). Body mass index and prevalence of obesity changes among Kuwaitis. *European Journal of Clinical Nutrition*.

[36] Raman SR, Al-Halabi B, Hamdan E, Landry MD (2012). Prevalence and risk factors associated with self-reported carpal tunnel syndrome (CTS) among office workers in Kuwait. *BioMed Central Research Notes*.

[37] Babusik P, Bilal M, Duris I (2011). Nonalcoholic fatty liver disease of two ethnic groups in Kuwait: comparison of prevalence and risk factors. *Medical Principles and Practice*.

[38] Al Zenki S, Al Omirah H, Al Hooti S (2012). High prevalence of metabolic syndrome among Kuwaiti adults–a wake-up call for public health intervention. *International Journal of Environmental Research and Public Health*.

[39] Al-Shaibani H, El-Batish M, Sorkhou I (2004). Prevalence of insulin resistance syndrome in a primary health care center in Kuwait. *Family Medicine*.

[40] Al-Isa AN (2004). Factors associated with overweight and obesity among Kuwaiti kindergarten female teachers. *Nutrition and Health*.

[41] Ramadan J, Barac-Nieto M (2003). Reported frequency of physical activity, fitness, and fatness in Kuwait. *American Journal of Human Biology*.

[42] Al-Isa AN (2003). Are Kuwaitis getting fatter?. *Nutrition and Health*.

[43] Jackson RT, Al-Mousa Z, Al-Raqua M, Prakash P, Muhanna AN (2002). Multiple coronary risk factors in healthy older Kuwaiti males. *European Journal of Clinical Nutrition*.

[44] Jackson RT, Al-Mousa Z, Al-Raqua M, Prakash P, Muhanna A (2001). Prevalence of coronary risk factors in healthy adult Kuwaitis. *International Journal of Food Sciences and Nutrition*.

[45] Al-Isa AN (1999). Dietary and socio-economic factors associated with obesity among Kuwaiti college men. *British Journal of Nutrition*.

[46] Al-Isa AN (1998). Factors associated with overweight and obesity among Kuwaiti college women. *Nutrition and Health*.

[47] Al-Isa AN (1997). Changes in body mass index (BMI) and prevalence of obesity among Kuwaitis 1980–1994. *International Journal of Obesity*.

[48] Al-Isa AN (1997). Changes in body mass index and prevalence of obesity among adult Kuwaiti women attending health clinics. *Annals of Saudi Medicine*.

[49] Al-Isa AN (1997). Temporal changes in body mass index and prevalence of obesity among Kuwaiti men. *Annals of Nutrition and Metabolism*.

[50] Al-Isa AN (1995). Prevalence of obesity among adult Kuwaitis: a cross-sectional study. *International Journal of Obesity and Related Metabolic Disorders*.

[51] Al-Awadi F, Amine EK (1989). Overweight and obesity in Kuwait. *Journal of the Royal Society of Health*.

[52] Alarouj M, Bennakhi A, Alnesef Y, Sharifi M, Elkum N (2013). Diabetes and associated cardiovascular risk factors in the state of Kuwait: the first national survey. *International Journal of Clinical Practice*.

[53] AlMajed HT, AlAttar AT, Sadek AA (2011). Prevalence of dyslipidemia and obesity among college students in Kuwait. *Alexandria Journal of Medicine*.

[54] Al-Nesf Y, Kamel M, El-Shazly MK (2006). Kuwait STEPS, 2006. *Kuwait Ministry of Health*.

[55] Aw T-C, Zoubeidi T, Al-Maskari F, Blair I (2011). Challenges and strategies for quantitative and qualitative field research in the United Arab Emirates. *Asian Pacific Journal of Cancer Prevention*.

[56] Berger G, Peerson A (2009). Giving young Emirati women a voice: participatory action research on physical activity. *Health and Place*.

