# Supplementary material for: A Review of Adult Obesity Prevalence, Trends, Risk Factors, and Epidemiologic Methods in Kuwait
Source: J Obes. 2013 Dec 17;2013:378650. doi: 10.1155/2013/378650 (PMC3877637; doi:10.1155/2013/378650)
Supplement: Supplementary file 1 — Table S1: Additional characteristics of adult obesity epidemiologic studies in Kuwait. [file 378650.f1.pdf]

A review of adult obesity prevalence, trends, risk factors and epidemiologic methods in Kuwait

Stalo Karageorgi[1], Osama Alsmadi[1], Kazem Behbehani[2]
[1]Genome Centre, Dasman Diabetes Institute, Kuwait city, Kuwait
[2]Senior Management, Dasman Diabetes Institute, Kuwait city, Kuwait

Corresponding author: Stalo Karageorgi
Email: stalo.karageorgi@dasmaninstitute.org
Address: P.O.Box 1180, Dasman 15462, Kuwait

Table S1. Additional characteristics of adult obesity epidemiologic studies in Kuwait

First Author (Publication Year)	Obesity Category	List of all collected variables reported in study	Other anthropometric measures	Author affiliation (first and last)	Journal (Impact Factor)
Badr H. (2012)	Outcome.	Age, gender, marital status, cultural background (Bedouin, non-Bedouin), education, total monthly household income, depression symptoms score, diabetes mellitus, hypertension, cardiac diseases, osteoarthritis.	No.	Kuwait University, Globe Consultancy.	Gerontologist (IF 2011: 2.48).
Zaghloul S. (2012)	Outcome.	Gender, education, marital status, employment, occupation, monthly family income, BMI, waist circumference, dietary variables including energy intake, macronutrients (protein, carbohydrates, fat, n-3 and n-6 fatty acids, fibre, cholesterol), vitamins (A, E, C, D, thiamin, riboflavin, niacin, B6, B12, folate), minerals (Ca, Fe, Mg, P, Se, Na, Zn).	Waist circumference.	Egypt National Nutrition Institute, Univerity of Maryland USA.	Public Health Nutrition (IF 2011: 2.17).
Alattar A. (2012)	Risk factor (for impaired glucose regulation).	Gender, age, family history of hypertension, family history of diabetes, current smoker, BMI, waist circumference, hypertension diagnosis, systolic and diastolic blood pressure, fasting blood glusose, 2-hour postprandial blood glucose, Hb1A1, cholesterol, triglycerides.	Waist circumference.	Al-Amiri Hospital, Public Authority for Applied Education and Training.	Medical Principles and Practice (IF 2010: 1.07)
Al-Isa A. (2011)	Outcome.	Age, dental status, chronic disease, number of obese brothers, number of obese relatives, parental obesity, education, wife's education, last GPA, high school GPA, number of persons living at home, monthly family income, physical activity, practice sport, number of male children, number of female children, health status, dieting, no of times dieted, feeling tired, needing special nutrition program.	No.	Kuwait University, University of British Columbia Canada.	Asia-Pacific Journal of Public Health (IF 2011: 1.06).
Ahmed F. (2011)	Outcome.	Age, gender, education, BMI, smoking, exercise.	No.	Griffith University Australia, Kuwait Ministry of Health.	Public Health Nutrition (IF 2011: 2.17).
Babusik P. (2010)	Risk factor (for other atherosclerosis risk factors).	Age, gender, nationality, BMI, WC, HC, WHR, FBG, TC, HDL, LDL, TG, TC/HDL.	Waist circumference, hip circumference, waist-to-hip ratio.	Al Rashid Hospital, Comenius University Slovak Republic.	Medical Principles and Practice (IF: 1.07).
Al Rashdan I. (2010)	Outcome.	Age, gender, BMI, waist circumference, BP, fasting GLC, HBA1c, HDL, LDL, Triglycerides.	Waist circumference.	Chest Diseases Hospital of Ministry of Health, Kuwait University.	Angiology (IF 2011: 1.51).
Al-Kandari F. (2008)	Outcome.	Age, nationality, marital status, income, BMI, HPLP score.	No.	College of Nursing.	Nursing and Health Sciences (IF 2011: 0.68).
Al-Bader W. (2008)	Risk factor (for pulmonary ventilatory function).	BMI, age, gender. height, waist to hip ratio, FVC, FEV.	Waist circumference, hip circumference, waist-to-hip ratio	Ministry of Public Health, Kuwait University.	Medical Principles and Practice (IF 2008: 0.54).

Table S1 *Continued*

First Author (Publication Year)	Obesity Category	List of all collected variables reported in study	Other anthropometric measures	Affiliation (first and last)	Journal (Impact Factor)
Al Orifan F. (2007)	Risk factor (for other cardiovascular risk factors).	Age, gender, smoking, physical activity, blood pressure, BMI, fasting blood glucose, TC, TG, HDL, LDL.	No.	Ministry of Health.	Kuwait Medical Journal (IF not available).
Al-Kandari Y. (2006)	Outcome.	BMI, waist circumference, skinfold thickness, age, education, annual income, occupation, SES, physical activity, number of children, number of relatives in same household, number of families in same household, degree of religiosity (memorized parts of Quran, gave alms to poor, attended religious lectures, performed extra religious activities, prayed the five prayers on time), number of times eating snacks between major meals, number of times eating in restaurant weekly, degree of preferring salt in food, having a cook, smoking, having separate kitchen for cooking.	Waist circumference, hip circumference, waist-to-hip ratio, triceps skinfold thickness, subscapular skinfold thickness.	Kuwait University.	Obesity Reviews (IF 2007: 7.82).
Al-Assomi F. (2005)	Outcome.	Age, sex, nationality, marital status, education, number of children, smoking, BMI, blood pressure, cholesterol, triglycerides, blood glucose, diabetes.	No.	Surra Clinic, Kuwait University.	Journal of the Bahrain Medical Society (IF not available).
Al-Shayji I. (2004)	Risk factor (for metabolic syndrome parameters).	Age, gender, smoking, current medication, family history of diabetes, hypertension, coronary heart disease, hyperlipidaemia, menopausal status, physical activity, blood pressure, BMI, waist and hip circumference, glucose, TG, TC, HDL, uric acid, LDL, apo B, serum insulin concentration.	Waist circumference, hip circumference.	Kuwait University.	Annals of Nutrition and Metabolism (IF 2004: 1.07)
Al-Asi T. (2003)	Outcome.	Age, gender, nationality, physical activity, diabetes, BMI, hypertension, type of work (field, office).	No.	Ahmadi Hospital.	Occupational Medicine (IF 2003: 0.69).
Olusi S. (2003)	Outcome.	Age, gender, education, monthly income, physical inactivity, BMI, diabetes, smoking, cholesterol, glucose, Apo-A/B.	No.	Kuwait University.	Annals of Saudi Medicine (IF 2003:0.12).
Al-Isa A. (1999)	Outcome.	Gender, age, marital status, obesity among parents, dieting, last health check-up, year of study in college, number of brothers, number of sisters, high school GPA, number of regular meals eaten.	No.	Kuwait University.	The Journal of the Royal Society for the Promotion of Health (IF: 0.41).
Abdella N. (1998)	Risk factor (for diabetes).	Age, gender, diabetes, education, exercise, parental history of diabetes, hypertension, BMI, fasting plasma glucose, cholesterol, triglycerides, uric acid, blood pressure.	No.	Kuwait University.	Diabetes Research and Clinical Practice (IF 1999: 0.69).
Al-Isa A. (1997)	Outcome.	Age, gender, governorate region, education, marital status, occupation.	No.	Kuwait University.	European Journal of Clinical Nutrition (IF 1999: 1.67).
